# Real estate data to analyse the relationship between property prices, sustainability levels and socio-economic indicators

**DOI:** 10.1016/j.dib.2020.106359

**Published:** 2020-10-06

**Authors:** Franz Fuerst, Michel Ferreira Cardia Haddad

**Affiliations:** a Department of Land Economy, University of Cambridge, Cambridge, CB3 9EP, United Kingdom

**Keywords:** Energy performance certificate, Hedonic pricing, Index of multiple deprivation, Real estate, Sustainability

## Abstract

Recent studies have sought to explore the relationship between environmental and financial performance, in particular the relationship between the energy efficiency level of a building and its financial value. The present real estate dataset contains 43 variables of repeat sales transactions, energy performance certificate (EPC) rating, index of multiple deprivation (IMD), and geographical location of properties in England and Wales involved in a total of 4,201 transactions from 1995 to 2012. This dataset enables researchers and practitioners to further explore important questions regarding the nexus between the real estate industry, sustainability levels, and socio-economic aspects. Due to the scarcity of publicly available quality real estate data, the dataset detailed in this article may play a relevant role by becoming easily discoverable, clearly explained, and structured to be ready to be used by researchers, analysts, and policymakers. The empirical analysis of the economic case for energy-efficient dwellings in the UK private rental market performed in Fuerst, et al. [1] is based on this dataset.

## Specifications Table

**Table:** 

Subject	Economics and Finance
Specific subject area	Renewable Energy, Sustainability and the Environment
Type of data	Table
How data were acquired	The data is publicly available from UK governmental sources
Data format	Raw, analysed/derived
Parameters for data collection	As the focus of this dataset is on repeat sales in the housing market, then an original larger dataset (which source is the Her Majesty's Land Registry), with all property sale transactions, was filtered to select only the cases in which the property was sold no less than two times, where at least one of the transactions was recorded after August 2008 (when EPCs became mandatory for residential properties in the United Kingdom). All remaining observations were excluded from the original dataset. Moreover, observations with incomplete data were discarded. There is no missing data in this dataset.
Description of data collection	Data were manual and directly extracted from the web address of each of the on-line data sources and, after data pre-treatments (e.g. filtering, merging), transformed into comma-separated values (CSV) file format
Data source location	Institutions: Her Majesty's Land Registry, Domestic Energy Performance Certificate Register, and Office for National Statistics (ONS) Country: United Kingdom
Data accessibility	Dataset supplied with this article paper
Related research article	Author's names: Franz Fuerst, Michel Ferreira Cardia Haddad, Hassan Adan Title: Is there an economic case for energy-efficient dwellings in the UK private rental market? Journal: Journal of Cleaner Production DOI: https://doi.org/10.1016/j.jclepro.2019.118642

## Value of the Data

• This dataset provides extensive information on residential sales transactions in England along with socio-economic indicators and property-level characteristics, notably on energy efficiency. While the underlying individual databases are mostly available in the public domain, this data adds value because it offers researchers an integrated ready-to-use dataset.
• Academic researchers will be able to glean important insights into the dynamics between property prices and other important features such as a geographical area's deprivation status or a property's energy efficiency level. Practitioners may apply this dataset to obtain market insights and/or for training purposes.
• A further possibility is the analysis of property price appreciation between two sales transactions to investigate how the market pricing of individual property features as well as sustainability and socio-economic indicators have changed during the study period.
• This dataset contains a number of additional derived variables for each property, ready to be used by researchers and practitioners, for example in hedonic pricing or other regression models.

## 1 Data Description

The present dataset focusses on a crucial industry of the economy (i.e. real estate), containing 43 variables related to 4,201 repeat sales transactions from 1995 to 2012, the respective energy performance certificate (EPC) rating, index of multiple deprivation (IMD), and geographical location of properties located in England and Wales. The empirical analysis of Fuerst, et al. [1] on the economic case for energy-efficient dwellings in the UK private rented sector (PRS) is based on this dataset. Rental data which were obtained from HomeCo Internet Property Ltd for analysing the effects of energy efficiency on PRS properties in that article are proprietary and, therefore, not considered here. Apart from this limitation, all relevant variables are described and made available with this article.

This dataset intends to provide relevant information to researchers and practitioners, allowing them to explore important questions involving the topics of real estate, sustainability levels, and socio-economic conditions. This dataset contains four variable groups (i.e. repeat sales transactions, EPC, IMD, and geographical location), which are detailed in following subsections. In total, there are 176,442 data points, excluding the identification variable (*id*), which variables are detailed in Table 1. There are no missing observations in this dataset.

**Table 1:** Details of each of the 43 variables in the dataset

Variable name (sic)	Variable context	Variable type	Data category	Data type	Description	Source
id	Identification	Derived	Categorical	Nominal	Identification number assigned to each transaction in this dataset	Own elaboration
price_1	Transactional	Raw	Numerical	Discrete	Property price, in pound sterling, paid in the first sale transaction	Her Majesty's Land Registry
date_1	Transactional	Raw	Categorical	Ordinal	Date (format: dd/mm/yyyy) of the first sale transaction	Her Majesty's Land Registry
price_2	Transactional	Raw	Numerical	Discrete	Property price, in pound sterling, paid in the second sale transaction	Her Majesty's Land Registry
date_2	Transactional	Raw	Categorical	Ordinal	Date (format: dd/mm/yyyy) of the second sale transaction	Her Majesty's Land Registry
perc_change_p2_to_p1	Transactional	Derived	Numerical	Continuous	Change, in percent, from the property price paid in the first (*price_1*) to the second (*price_2*) sale transaction	Own elaboration
days_between_sale	Transactional	Derived	Numerical	Discrete	Period of time, in days, from the first (*date_1*) to the second (*date_2*) sale transaction	Own elaboration
ln_price_1	Transactional	Derived	Numerical	Continuous	Natural logarithm of the variable *price_1*	Own elaboration
ln_price_2	Transactional	Derived	Numerical	Continuous	Natural logarithm of the variable *price_2*	Own elaboration
epc_100	Sustainability	Raw	Numerical	Discrete	Standard assessment procedure (SAP) points assigned to the property, ranging from 1 to 100 (where 1 is the least efficient)	Ministry of Housing, Communities & Local Government
epc_rating_a	Sustainability	Raw	Categorical	Boolean	Property with EPC rating assigned as band A (92-100 SAP points) when the value is 1 and 0 otherwise	Ministry of Housing, Communities & Local Government
epc_rating_b	Sustainability	Raw	Categorical	Boolean	Property with EPC rating assigned as band B (81-91 SAP points) when the value is 1 and 0 otherwise	Ministry of Housing, Communities & Local Government
epc_rating_c	Sustainability	Raw	Categorical	Boolean	Property with EPC rating assigned as band C (69-80 SAP points) when the value is 1 and 0 otherwise	Ministry of Housing, Communities & Local Government
epc_rating_d	Sustainability	Raw	Categorical	Boolean	Property with EPC rating assigned as band D (55-68 SAP points) when the value is 1 and 0 otherwise	Ministry of Housing, Communities & Local Government
epc_rating_e	Sustainability	Raw	Categorical	Boolean	Property with EPC rating assigned as band E (39-54 SAP points) when the value is 1 and 0 otherwise	Ministry of Housing, Communities & Local Government
epc_rating_f	Sustainability	Raw	Categorical	Boolean	Property with EPC rating assigned as band F (21-38 SAP points) when the value is 1 and 0 otherwise	Ministry of Housing, Communities & Local Government
epc_rating_g	Sustainability	Raw	Categorical	Boolean	Property with EPC rating assigned as band G (1-20 SAP points) when the value is 1 and 0 otherwise	Ministry of Housing, Communities & Local Government
ln_epc_100	Sustainability	Derived	Numerical	Continuous	Natural logarithm of the variable *epc_100*	Own elaboration
imd_score	Socio-economic	Raw	Numerical	Discrete	Index of multiple deprivation (IMD) rank (where 1 is most deprived) assigned to the property	Ministry of Housing, Communities & Local Government
imd_level	Socio-economic	Raw	Numerical	Discrete	Index of multiple deprivation (IMD) decile (where 1 is most deprived 10% of LSOAs) assigned to the property	Ministry of Housing, Communities & Local Government
income_score	Socio-economic	Raw	Numerical	Discrete	Income deprivation rank (where 1 is most deprived) assigned to the property	Ministry of Housing, Communities & Local Government
income_level	Socio-economic	Raw	Numerical	Discrete	Income deprivation decile (where 1 is most deprived 10% of LSOAs) assigned to the property	Ministry of Housing, Communities & Local Government
emp_score	Socio-economic	Raw	Numerical	Discrete	Employment deprivation rank (where 1 is most deprived) assigned to the property	Ministry of Housing, Communities & Local Government
emp_level	Socio-economic	Raw	Numerical	Discrete	Employment deprivation decile (where 1 is most deprived 10% of LSOAs) assigned to the property	Ministry of Housing, Communities & Local Government
educ_score	Socio-economic	Raw	Numerical	Discrete	Education skills and training deprivation rank (where 1 is most deprived) assigned to the property	Ministry of Housing, Communities & Local Government
educ_level	Socio-economic	Raw	Numerical	Discrete	Education skills and training deprivation decile (where 1 is most deprived 10% of LSOAs) assigned to the property	Ministry of Housing, Communities & Local Government
health_score	Socio-economic	Raw	Numerical	Discrete	Health deprivation and disability rank (where 1 is most deprived) assigned to the property	Ministry of Housing, Communities & Local Government
health_level	Socio-economic	Raw	Numerical	Discrete	Health deprivation and disability decile (where 1 is most deprived 10% of LSOAs) assigned to the property	Ministry of Housing, Communities & Local Government
crime_score	Socio-economic	Raw	Numerical	Discrete	Crime rank (where 1 is most deprived) assigned to the property	Ministry of Housing, Communities & Local Government
crime_level	Socio-economic	Raw	Numerical	Discrete	Crime decile (where 1 is most deprived 10% of LSOAs) assigned to the property	Ministry of Housing, Communities & Local Government
barrier_score	Socio-economic	Raw	Numerical	Discrete	Barriers to housing and services rank (where 1 is most deprived) assigned to the property	Ministry of Housing, Communities & Local Government
barrier_level	Socio-economic	Raw	Numerical	Discrete	Barriers to housing and services decile (where 1 is most deprived 10% of LSOAs) assigned to the property	Ministry of Housing, Communities & Local Government
living_score	Socio-economic	Raw	Numerical	Discrete	Living environment deprivation rank (where 1 is most deprived) assigned to the property	Ministry of Housing, Communities & Local Government
living_level	Socio-economic	Raw	Numerical	Discrete	Living environment deprivation decile (where 1 is most deprived 10% of LSOAs) assigned to the property	Ministry of Housing, Communities & Local Government
reg_north_east	Geography	Raw	Categorical	Boolean	Property located in the North East region when the value is 1 and 0 otherwise	Office for National Statistics
reg_north_west	Geography	Raw	Categorical	Boolean	Property located in the North West region when the value is 1 and 0 otherwise	Office for National Statistics
reg_yorkshire_and_the_humber	Geography	Raw	Categorical	Boolean	Property located in the Yorkshire and the Humber region when the value is 1 and 0 otherwise	Office for National Statistics
reg_east_midlands	Geography	Raw	Categorical	Boolean	Property located in the East Midlands region when the value is 1 and 0 otherwise	Office for National Statistics
reg_west_midlands	Geography	Raw	Categorical	Boolean	Property located in the West Midlands region when the value is 1 and 0 otherwise	Office for National Statistics
reg_east_of_england	Geography	Raw	Categorical	Boolean	Property located in the East of England region when the value is 1 and 0 otherwise	Office for National Statistics
reg_london	Geography	Raw	Categorical	Boolean	Property located in the London region when the value is 1 and 0 otherwise	Office for National Statistics
reg_south_east	Geography	Raw	Categorical	Boolean	Property located in the South East region when the value is 1 and 0 otherwise	Office for National Statistics
reg_south_west	Geography	Raw	Categorical	Boolean	Property located in the South West region when the value is 1 and 0 otherwise	Office for National Statistics

The dataset is prepared to facilitate future analyses. Six variables are derived/ calculated from raw variables, such as the natural logarithm (log henceforth) of transaction prices, the log of the standard assessment procedure (SAP) points assigned to each property, and the difference in days from the first to the second sale transaction. Moreover, the variables regarding the EPC band and geographical location are transformed into Boolean variables for usability purposes (e.g. regression analysis estimation).

### 1.1 Repeat sales transactions variables

There are eight variables detailing each of the 4,201 repeat sales transactions, being four raw and four derived variables, out of which six are numerical and two are categorical (i.e. date) variables. The four raw variables consist of the prices (in pound sterling) and dates of the first and second property sale transactions. Moreover, the four derived variables refer to the natural log of the prices of the first and second property sale transactions, the percentual change between such prices, and the time length (in days) between both transactions. The descriptive statistics of the six numerical variables are reported in Table 2.

**Table 2:** Descriptive statistics of the numerical variables directly related to the property sale transactions

Variable	Mean	Median	Std. Dev.	Skewness	Kurtosis	Smallest	Largest	Obs	Normal
price_1	120,190.90	100,000.00	189,750.50	23.97	693.03	6,000.00	5,660,000.00	4,201	0.00001
price_2	154,575.30	120,000.00	263,755.50	24.50	709.91	25,000.00	7,900,000.00	4,201	0.00001
ln_price_1	11.46	11.51	0.65	-0.01	4.65	8.70	15.55	4,201	0.00001
ln_price_2	11.75	11.70	0.52	1.16	8.02	10.13	15.88	4,201	0.00001
perc_change_p2_to_p1	0.50	0.20	0.83	3.13	22.93	-0.62	10.42	4,201	0.00001
days_between_sale	2,400.08	2,196.00	1,236.92	0.56	2.68	187.00	6,156.00	4,201	0.00001

Note: the header described as ‘Normal’ refers to the Shapiro-Francia normality test. The null hypothesis is that the data follows a Gaussian distribution.

**Fig. 1:**
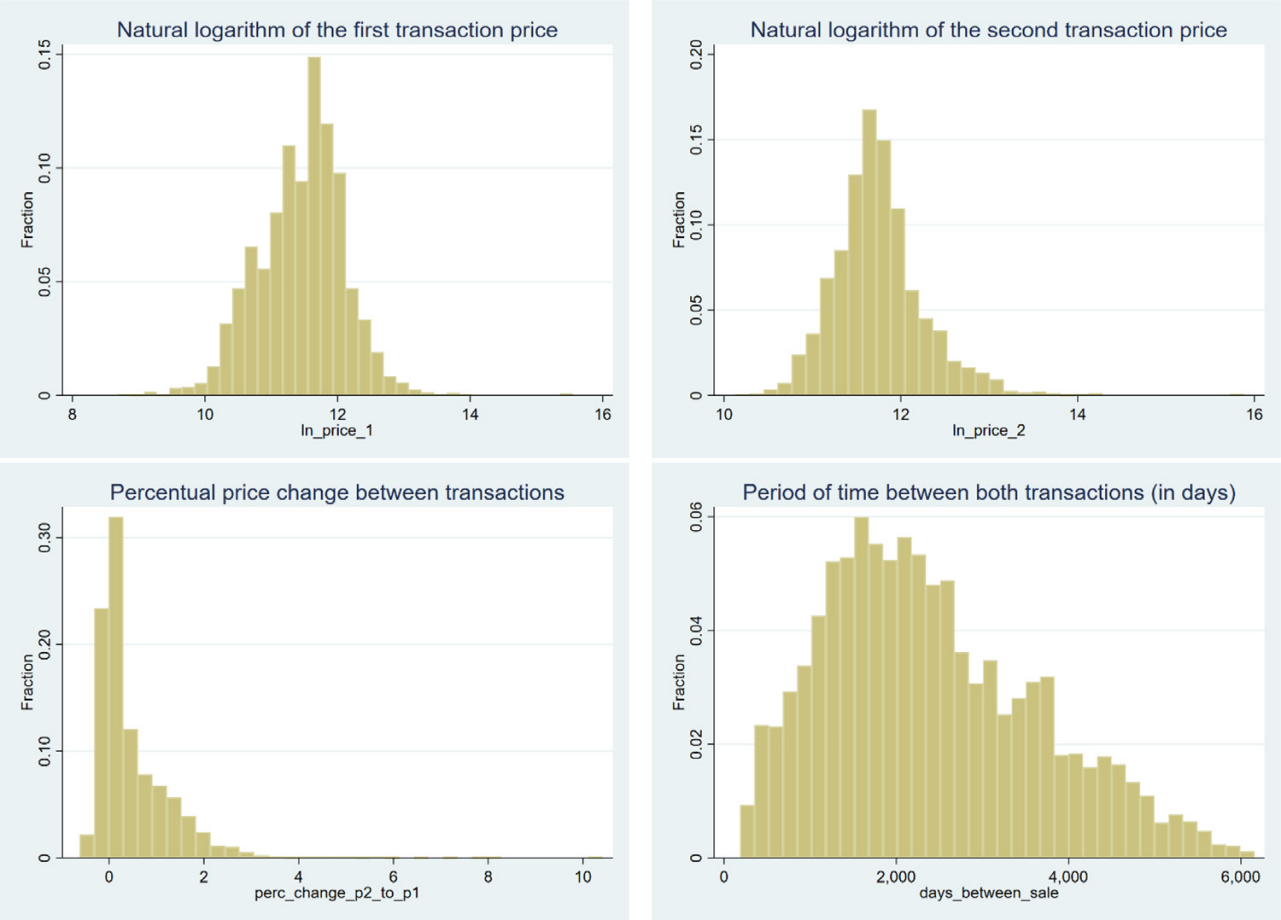
Distributions of the log price paid in the first (upper left hand side) and second (upper right hand side) property sale transactions, price variation between the first and second sale transaction (bottom left hand side), and period of time from the first to the second transaction (bottom right hand side)

Source: own elaboration

In summary, the statistics in Table 2 show that the log price of the first transaction (*ln_price_1*) is the only variable with negative skewness, although it is almost negligible. Moreover, all variables have kurtosis above three, except by the *days_between_sale* variable. Such data characteristics are depicted in Fig. 1, in which histograms confirm the descriptive statistics reported in Table 2.

In addition, the histograms shown in Fig. 1 confirm, through a data visualisation approach, the results of the Shapiro-Francia normality tests reported in Table 2, that these variables are not drawn from a normal distribution.

### 1.2 Energy performance certificate (EPC) variables

An energy performance certificate (EPC) consists of a rating measure that seeks to succinctly describe energy efficiency levels of real estate properties in the European Union. In the year of 2008, this measurement system is adopted by England and Wales. Based on such a rating measure, there are seven EPC bands, ranging from band A (i.e. the most efficient) to band G (i.e. the least efficient) [2]. Moreover, an EPC must be provided by the landlord before a property may be rented or sold [3], [4].

In the present dataset there are nine variables related to EPC, out of which seven are Boolean variables. In terms of EPC ratings based on the standard assessment procedure (SAP) points, Table 3 shows that the majority of the properties involved in the repeat sales transactions are assigned as EPC bands C and D, corresponding to almost 70% of the total in the dataset. The third EPC band with most cases is band E, totalling almost 700 properties (i.e. around 17% of the dataset), followed by band B, with almost 10% of the dataset. In addition, in this dataset there is no property assigned as band A, and approximately 5% of the properties are assigned to the two least efficient bands (i.e. F and G), totalling around 200 properties.

**Table 3:** Frequency and fraction of the seven EPC bands

EPC band	Frequency	Fraction
EPC A	0	0.00
EPC B	379	0.09
EPC C	1,442	0.34
EPC D	1,480	0.35
EPC E	699	0.17
EPC F	162	0.04
EPC G	39	0.01
Total	4,201	1.00

**Fig. 2:**
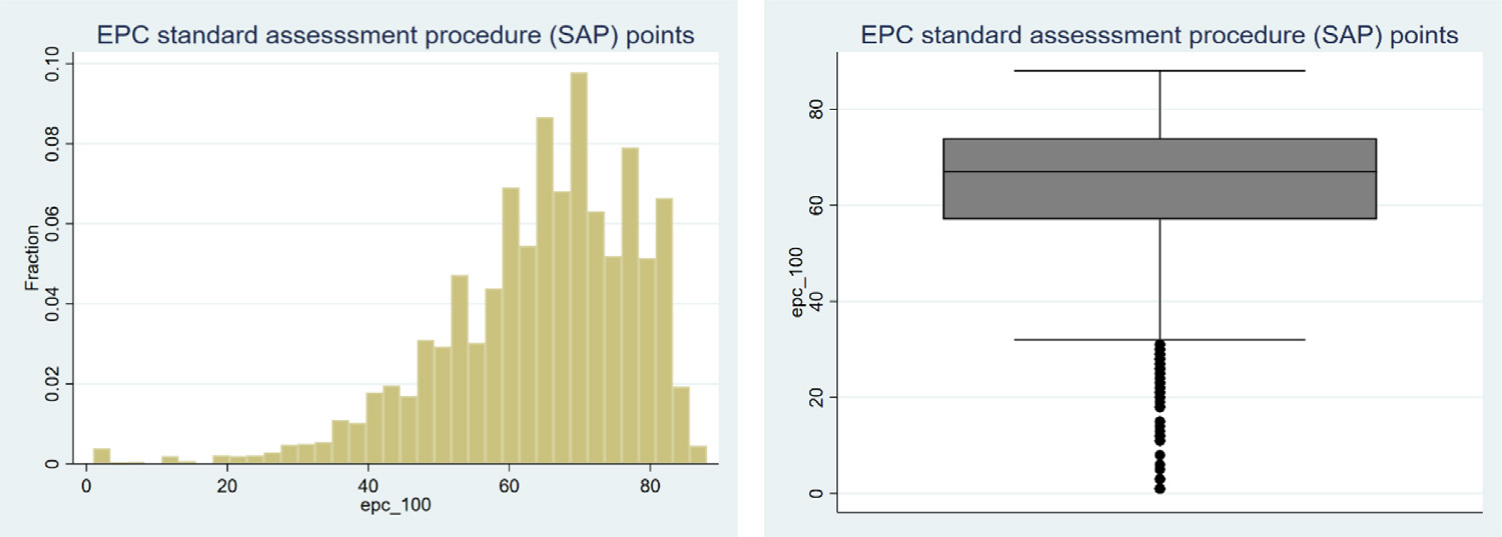
Histogram (left hand side) and box plot (right hand side) of the distribution of the SAP points

Source: own elaboration

By breaking down the EPC band classification in terms of the SAP points, it is possible to realise that most cases are within the range between 60 and 80 SAP points, totalling 2,520 observations, corresponding to 60% of the cases in the dataset, as depicted in Fig. 2.

In terms of potential outliers, the box plot in Fig. 2 shows that there are few observations containing properties with SAP points assigned below the value of 37. More precisely, there are 150 cases (4% of the dataset) below two standard deviations from the mean of 64 points, 47 cases (1% of the dataset) below three standard deviations from the mean, and still nine cases (0.5% of the dataset) below four standard deviations from the mean.

### 1.3 Index of multiple deprivation (IMD) variables

**Fig. 3:**
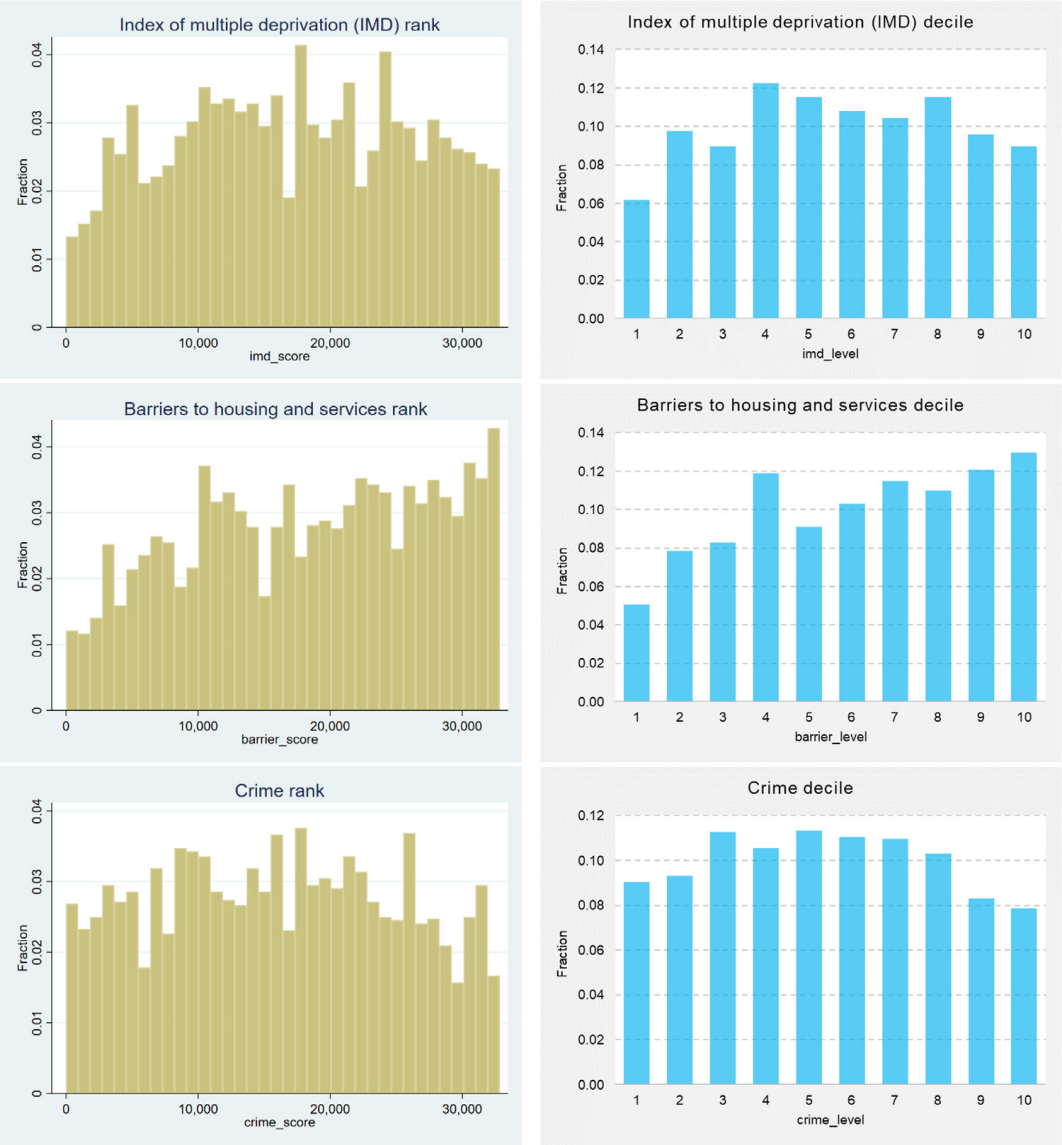

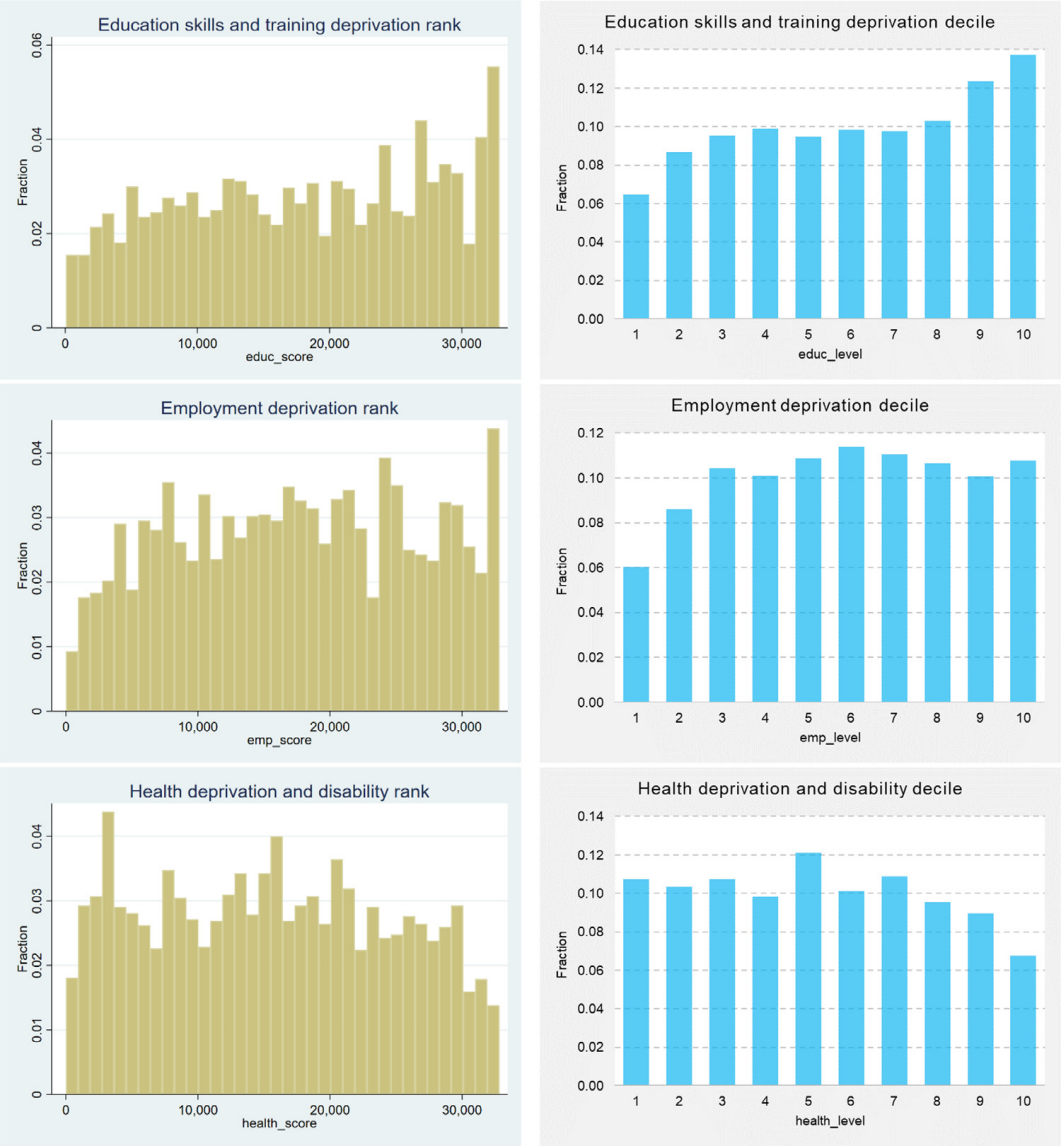

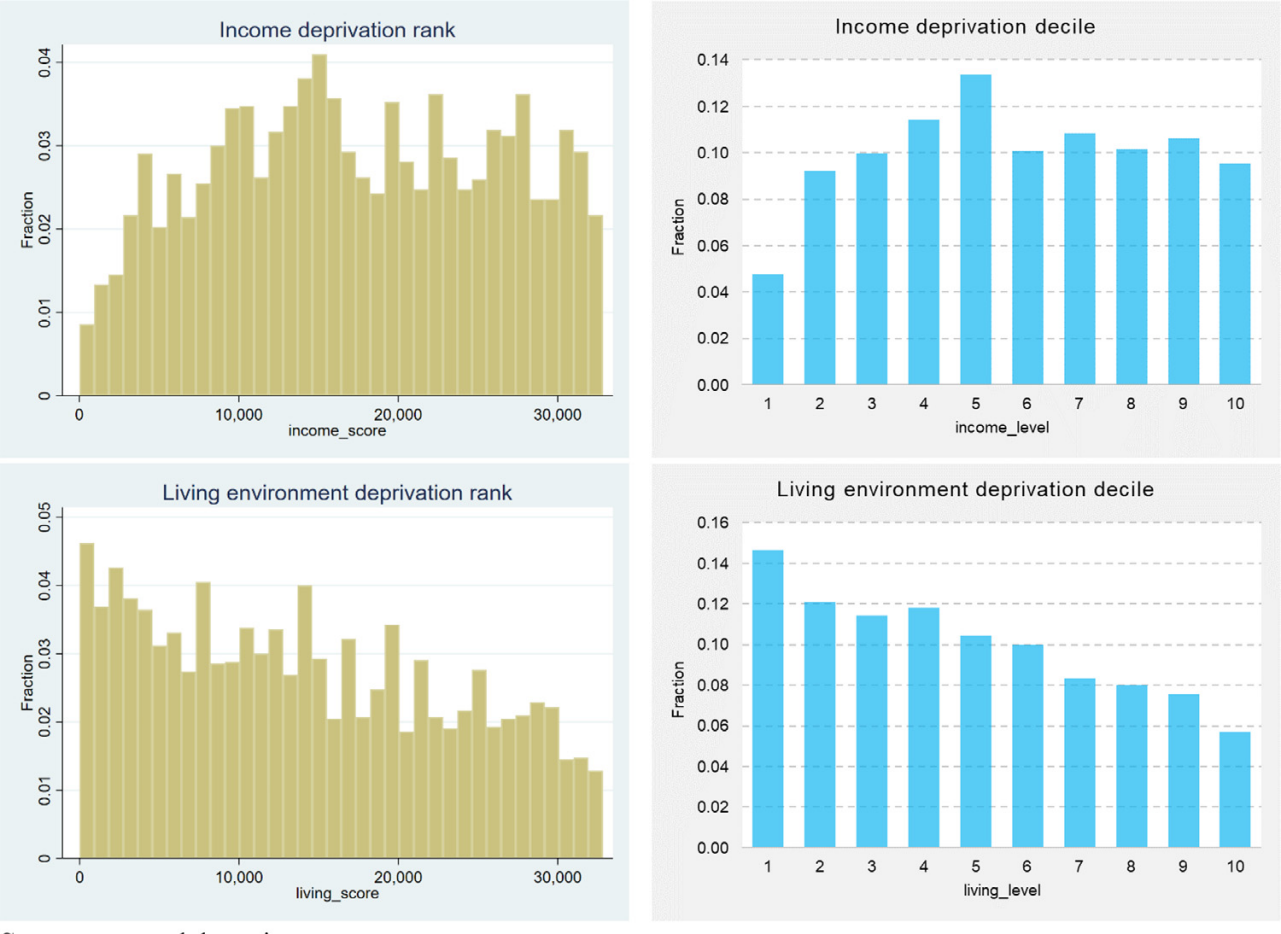
Histograms (left hand side) and bar charts (right hand side) of the IMD and its seven domains, considering their ranks and deciles, respectively

Source: own elaboration

The index of multiple deprivation (IMD) is used in the United Kingdom to rank relative deprivation levels assigned to each of the geographical locations classified as a Lower Layer Super Output Area (LSOA). Besides the IMD – which is an overall measure, area characteristics based on the LSOA levels include seven domains, consisting of barriers to housing and services, crime, quality of schooling (i.e. education, skills and training), employment, health and disability, income, and living environment [5]. Dwellings located in the 10% most deprived neighbourhoods are in the bottom decile (i.e. IMD decile equals to 1 in Fig. 3) and, conversely, those in the 10% least deprived neighbourhoods are in the top decile (i.e. IMD decile equals to 10 in Fig. 3).

In general, the properties in the dataset appear to be distributed roughly evenly across all overall deprivation levels. However, it is worth noting that the first IMD decile has considerably fewer absolute cases compared to the remaining respective deciles, as depicted in Fig. 3. Approximately 6% of properties are in the worst IMD deprivation Level 1 and, conversely, 9% of properties are in the decile of least deprived neighbourhoods.

In both cases (i.e. the IMD and its seven domains), the data are measured in two units, consisting of deprivation ranks/ scores and also respective deciles/ levels, affording the analyst a greater level of flexibility and freedom for data reusability purposes. Regarding the remaining seven domains values, properties from all deprivation levels (i.e. from 1 to 10) are included in the dataset, which is a relevant aspect from a data and sample representativeness point of view.

### 1.4 Geographical location variables

The geographical distribution of the properties involved in the repeat sales transactions included in this dataset follows the classification adopted by the Office for National Statistics (ONS), totalling nine regions (formerly kwon as ‘government offices for the regions’ or GOR). The regions with most transactions are North West, Yorkshire and The Humber, and West Midlands, which combined correspond to around 54% of the transactions in the dataset, as reported in Table 4. Conversely, the regions with the least number of transactions are South West, East of England, and North East, which combined represent less than 20% of the transactions in the dataset.

**Table 4:** Geographical distribution of the transactions included in the dataset

Geography	Transactions frequency	Transactions fraction	Population fraction
North West	840	0.20	0.13
Yorkshire and The Humber	839	0.20	0.10
West Midlands	599	0.14	0.11
East Midlands	435	0.10	0.09
South East	407	0.10	0.16
London	361	0.09	0.16
South West	287	0.07	0.10
East of England	279	0.07	0.11
North East	154	0.04	0.05
Total	4,201	1.00	1.00

The geographical distribution of the dataset is also compared with the population distribution of England and Wales [6]. Despite some disparities, the distribution of the transactions in the dataset may be considered as a representative sample.

## 2 Experimental Design, Materials and Methods to Acquire the Data

**Fig. 4:**
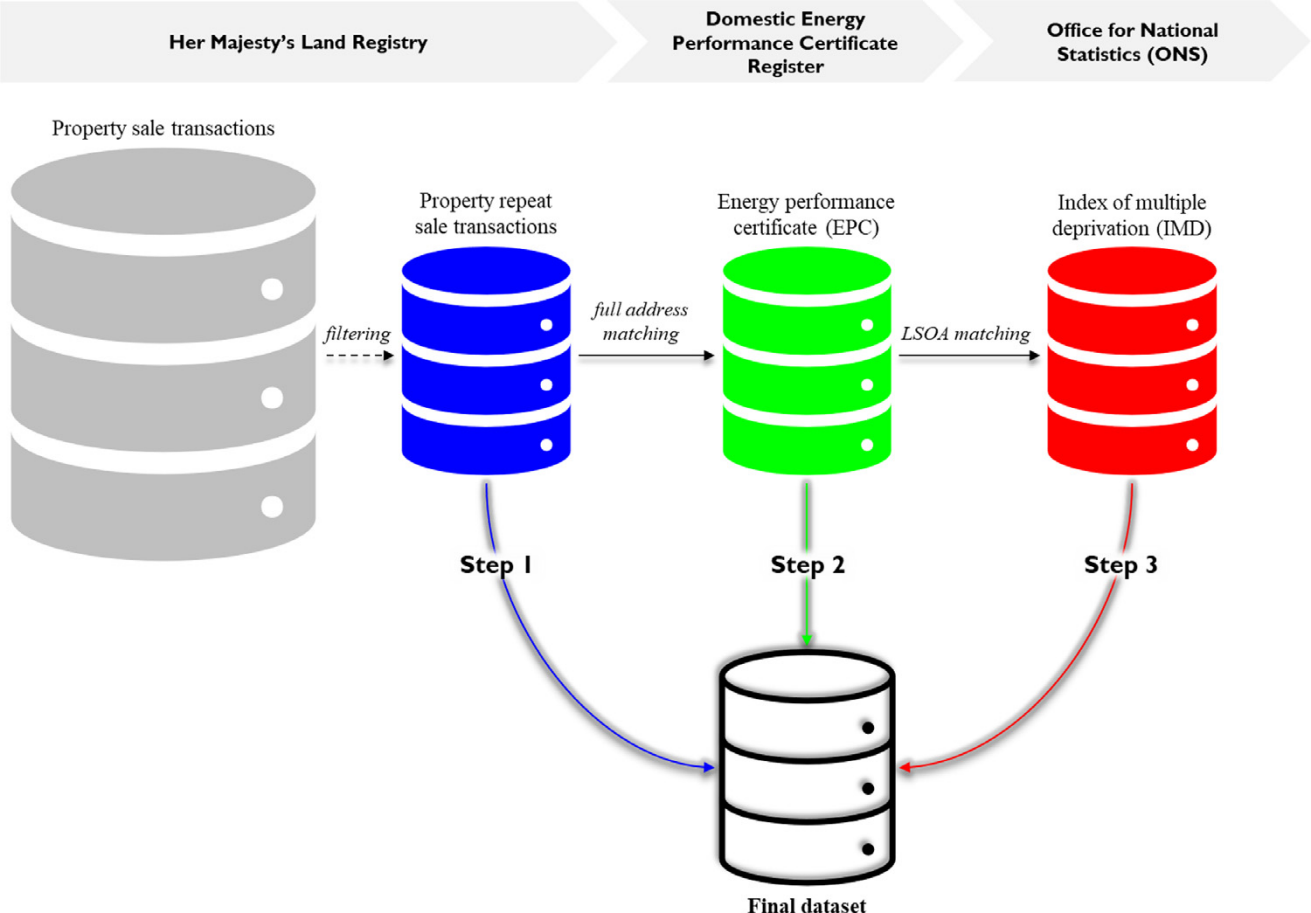
Flowchart of the protocol for data collection

Source: own elaboration

Data from a variety of publicly available sources are extracted, filtered, and merged through a three step process, following the protocol for data collection depicted in Fig. 4. In the first step, data on market prices and transaction dates are manually extracted from the Her Majesty's Land Registry on-line database, comprising residential transaction prices submitted in the period between 1995 and 2012. A filter is applied to this larger dataset (with all property sales transactions) to select only properties that were sold at least twice, in which at least one of the transactions is recorded after August 2008, when EPCs became mandatory for residential properties in the United Kindgom.

In the second step, information related to the EPC is manually extracted from the Domestic Energy Performance Certificate Register (under the Ministry of Housing, Communities & Local Government) on-line database, which is then merged, through full address matching, with the dataset previously produced in the first step.

In the third step, the dataset is enhanced by adding socio-economic data (i.e. the IMD and its seven domains), which are manually extracted from the ONS postcode directory and then subsequenty merged, through LSOA matching, with the dataset previously produced in the second step. In order to ensure a representative sample, observations across hundreds of different neighbourhoods in England and Wales are obtained via a stratified random draw.

## Declaration of Competing Interest

The authors declare no competing financial interests or any other interests that might be perceived to influence the results and/or discussion reported in this data article.

## Appendix Supplementary materials

Image, application 1

Image, application 2
